# Candidate gene discovery of *Botrytis cinerea* resistance in grapevine based on QTL mapping and RNA-seq

**DOI:** 10.3389/fpls.2023.1127206

**Published:** 2023-02-07

**Authors:** Kai Su, Wei Zhao, Hong Lin, Changyue Jiang, Yuhui Zhao, Yinshan Guo

**Affiliations:** ^1^ College of Horticulture, Shenyang Agricultural University, Shenyang, China; ^2^ College of Horticulture Science and Technology, Hebei Normal University of Science and Technology, Qinhuangdao, China; ^3^ Hebei Key Laboratory of Horticultural Germplasm Excavation and Innovative Utilization, Qinhuangdao, China; ^4^ National & Local Joint Engineering Research Center of Northern Horticultural Facilities Design and Application Technology (Liaoning), Shenyang, China

**Keywords:** *Vitis vinifera*, gray mold, resistance breeding, transcriptome analysis, QTL mapping

## Abstract

Grape gray mold disease (*Botrytis cinerea*) is widespread during grape production especially in *Vitis vinifera* and causes enormous losses to the grape industry. In nature, the grapevine cultivar ‘Beta ‘ (*Vitis riparia* × *Vitis labrusca*) showed high resistance to grape gray mold. Until now, the candidate genes and their mechanism of gray mold resistance were poorly understood. In this study, we firstly conducted quantitative trait locus (QTL) mapping for grape gray mold resistance based on two hybrid offspring populations that showed wide separation in gray mold resistance. Notably, two stable QTL related to gray mold resistance were detected and located on linkage groups LG2 and LG7. The phenotypic variance ranged from 6.86% to 13.70% on LG2 and 4.40% to 11.40% on LG7. Combined with RNA sequencing (RNA-seq), one structural gene *VlEDR2* (Vitvi02g00982) and three transcription factors *VlERF039* (Vitvi00g00859), *VlNAC047* (Vitvi08g01843), and *VlWRKY51* (Vitvi07g01847) that may be involved in *VlEDR2* expression and grape gray mold resistance were selected. This discovery of candidate gray mold resistance genes will provide an important theoretical reference for grape gray mold resistance mechanisms, research, and gray mold-resistant grape cultivar breeding in the future.

## 1 Introduction

*Vitis vinifera* L. belongs to genus *Vitis* of the family Vitaceae. As a major table grape resource, it possesses important social and economic values in the world. While in China, due to the temperate continental climate, it is easily infected by many fungal diseases among which the grape gray mold that is caused by *Botrytis cinerea* Pers. was one of the major pathogens (Choquer et al., 2007). In most grape production regions, in case of infection by grape gray mold, the yield would reduce by 20%–60%, and the berry quality would also face huge damages (Martínez-Romero et al., 2007; Dean et al., 2012; Saito et al., 2019). During the grape production process, antifungal agents could inhibit the occurrence of diseases to a certain extent, but this is not recommended due to environmental pollution and food safety. At present, the breeding of high gray mold resistance grapevine cultivar became a hot point. In nature, many grapevine resources possess higher gray mold resistance than *V. vinifera* L., including *Vitis amurensis* Rupr., *Vitis quinquangularis* Rehd., *Vitis piasezkii* Maxim., *Vitis riparia* Michx, *Vitis rupestris* Scheele, and *Vitis labrusca* L. (Gabler et al., 2003; Wan et al., 2015).

Marker-assisted selection based on genetic linkage map construction and quantitative trait locus (QTL) mapping has been widely used to screen high disease resistance grapevine cultivars through traditional crossbreeding strategies such as ripe rot, downy mildew, powdery mildew, and white rot (Barba et al., 2014; Teh et al., 2017; Fu et al., 2019; Sapkota et al., 2019; Tello et al., 2019; Su et al., 2021) for its high breeding efficiency. Until now, there were no QTL mapping reports related to grape gray mold resistance, and research on gray mold resistance transcriptional regulation mechanism was majorly focused on the transcription factor ERF and MYB families in *Arabidopsis* and tomato (Lorenzo et al., 2003; Pre et al., 2008; Zhao et al., 2012; Liu et al., 2021). In grapevine, there have been some reports related to gray mold resistance including structure genes *VvSWEE4*, *VvSWEE15*, *VvSWEET7*, and *VvAMP2* and some transcription factors including *VvWRKY52*, *VqERF072*, *VqERF112*, *VqERF114*, *VaERF20*, *VaERF16*, and *VaMYB306* (Nanni et al., 2014; Jiao et al., 2015; Wang et al., 2018a; Wang et al., 2018b; Breia et al., 2019; Zhu et al., 2019; Wang et al., 2020; Zhu et al., 2022). While the quantitative trait was controlled by many genes, candidate genes related to gray mold resistance in grapevine still need to be explored.

In this study, we selected three gray mold resistance grapevine cultivars, ‘Zhuosexiang’ (‘ZSX’) (*V. vinifera* × *V. labrusca*), ‘Venus seedless’ (‘VS’) (*V. vinifera* × *V. labrusca*), and ‘Beta’ (“BT”) (*V. riparia* × *V. labrusca*), and two susceptible cultivars, ‘Red Globe’ (‘RG’) and ‘Victoria’ (‘VT’), which belong to *V. vinifera*. Among these grapevine cultivars, ‘RG’ was identified as one of the highly susceptible grape cultivars to *B. cinerea* (Wan et al., 2015), and ‘BT’ was usually used as rootstock for its high cold and disease resistance character. Based on the hybrid population and high-density genetic linkage map (Zhu et al., 2018; Su et al., 2021), which was created through interspecific crossing of ‘ZSX’ × ‘VT’ and ‘RG’ × ‘VS,’ we firstly conducted QTL mapping for gray mold resistance, and then transcriptome analysis was conducted for ‘RG’ and ‘BT’ at different infection stages on account of their most distinct resistance level of grapevine gray mold. Finally, candidate genes related to grapevine gray mold resistance were screened by QTL mapping and RNA sequencing (RNA-seq).

## 2 Materials and methods

### 2.1 Plant material and gray mold resistance identification

Grape cultivars ‘RG’ (*V. vinifera* L.), ‘VT’ (*V. vinifera* L.), ‘ZSX’ (*V. vinifera* × *V. labrusca*), ‘VS’ (*V. vinifera* × *V. labrusca*), and ‘Beta’ (‘BT’) (*V. riparia* × *V. labrusca*) and two hybrid populations were cultivated in the Grape Experimental Garden of Shenyang Agricultural University (23°24’N, 41°50’E), China. Interspecific hybridization of ‘RG’ × ‘VS’ was conducted in May 2009; ‘RG’ was used as the female parent, and ‘VS’ was used as the male parent. ‘ZSX’ × ‘VT’ was conducted in May 2014; ‘ZSX’ was used as the female parent, and ‘VT’ was used as the male parent. A total of 177 and 176 individuals from ‘RG’ × ‘VS’ and ‘ZSX’ × ‘VT’ were used for the gray mold resistance identification in 2019 and 2020. The third-to-fourth leaf from the tip of an annual branch was selected (three leaves per individual). The collected leaves were rinsed with 70% ethanol for 1 min, followed by 10% sodium hypochlorite for 1 min, and rinsed three times with ultrapure water. Next, the leaves were placed in plastic culture dishes and punctured in the left, middle, and right regions. Ten microliters of 10^7^/ml gray mold spore suspension was then dripped on the wound points to induce gray mold infection. Leaves with gray mold spores were incubated in a moist chamber at 28°C with 95% relative humidity. The lesion area of the infected region of each leaf was measured with a YMJ-C smart leaf area meter (Tuopu Instrument, Guangdong, China) (Su et al., 2021). Leaf samples of ‘RG’ and ‘BT’ that showed distinct resistance to gray mold at 0, 72, and 120 h after infection were collected for RNA-seq. Three biological replicates were collected at different infection periods of each cultivar with at least three leaves per replicate.

### 2.2 Gray mold resistance quantitative trait locus mapping

The lesion area (mean value of three replicates) of each genotype collected in 2019 and 2020 was used for QTL mapping. The integrated genetic linkage maps of ‘RG’ × ‘VS’ and ‘ZSX’ × ‘VT’ used in this research were constructed by using Restriction-site Associated DNA (RAD)-Sequencing, including 6,249 and 70,061 single nucleotide polymorphism (SNP) markers (Zhu et al., 2018; Su et al., 2021). A multiple QTL mapping (MQM) method was used to find significant QTL after a 1,000-permutation test (α = 0.05) based on the R/qtl package (Broman et al., 2003), and finally, the Logarithm of odds (LOD) threshold was set to 3. The max.qtl was set to 10 for forward selection. A 1-LOD confidence interval corresponding to the 95% confidence interval was calculated by using the “lodint” function. The explained phenotypic variation of each QTL phenotypic variation explained (PVE) was estimated using the “fitqtl” function. Candidate genes within the confidence interval of each QTL on the integrated map were selected according to 12X.v2 version of the Grape Genome database (https://urgi.versailles.inra.fr/Species/Vitis/Data-Sequences/Genome-sequences).

### 2.3 Gray mold resistance transcriptome analysis

RNA integrity was assessed using the RNA Nano 6000 Assay Kit and the Bioanalyzer 2100 system (Agilent Technologies, Santa Clara, CA, USA). The input material for the RNA sample preparation was 1-μg RNA per sample. Sequencing libraries were generated using the NEBNext^®^ Ultra™ RNA Library Prep Kit (New England Biolabs, Ipswich, MA, USA) and then sequenced on an Illumina Novaseq platform. Finally, 150-bp paired-end reads were generated. Clean reads were obtained by removing reads containing adapter, ploy-N, and low-quality reads from the raw data. The high-quality and paired-end clean reads were aligned to the reference genome (https://urgi.versailles.inra.fr/Species/Vitis/Data-Sequences/Genome-sequences) using HISAT 2v2.0.5 software, and the mapped reads of each sample were assembled by StringTie. The fragments per kilobase per million (FPKM) value of each gene was calculated based on the length of the gene and the number of reads mapped to this gene. Differential expression analysis was performed using the DESeq2 R package (1.20.0), and genes with an adjusted P-value <0.05 found by DESeq2 were assigned as differentially expressed. Gene Ontology (GO) enrichment analysis of differentially expressed genes (DEGs) was implemented by the clusterProfiler R package.

### 2.4 qRT-PCR validation of candidate genes

Infected leaves of grape cultivars ‘RG’ and ‘BT’ at 0, 72, and 120 h after gray mold infection were collected, and then these samples were used for total RNA extraction according to the manufacturer’s instructions of Plant Total RNA Isolation Kit (SK8631; Sangon Biotech, Shanghai, China). The PrimeScript™ RT-PCR Kit (RR047A; TaKaRa Bio, Kusatsu, Japan) was used to conduct cDNA synthesis, and the cDNA was diluted five times. Quantitative real-time PCR (qRT-PCR) was conducted in ABI QuantStudio™ 6 Flex System (Applied Biosystems). The relative expression level of selected genes was normalized to grapevine β-actin (Fujimori et al., 2016) and calculated using the 2^-ΔΔCT^ method. All reactions were performed using three biological replicates. The primers used in this study are listed in **Table S1**.

## 3 Results

### 3.1 Identification of grapevine gray mold resistance

**Figure 1:**
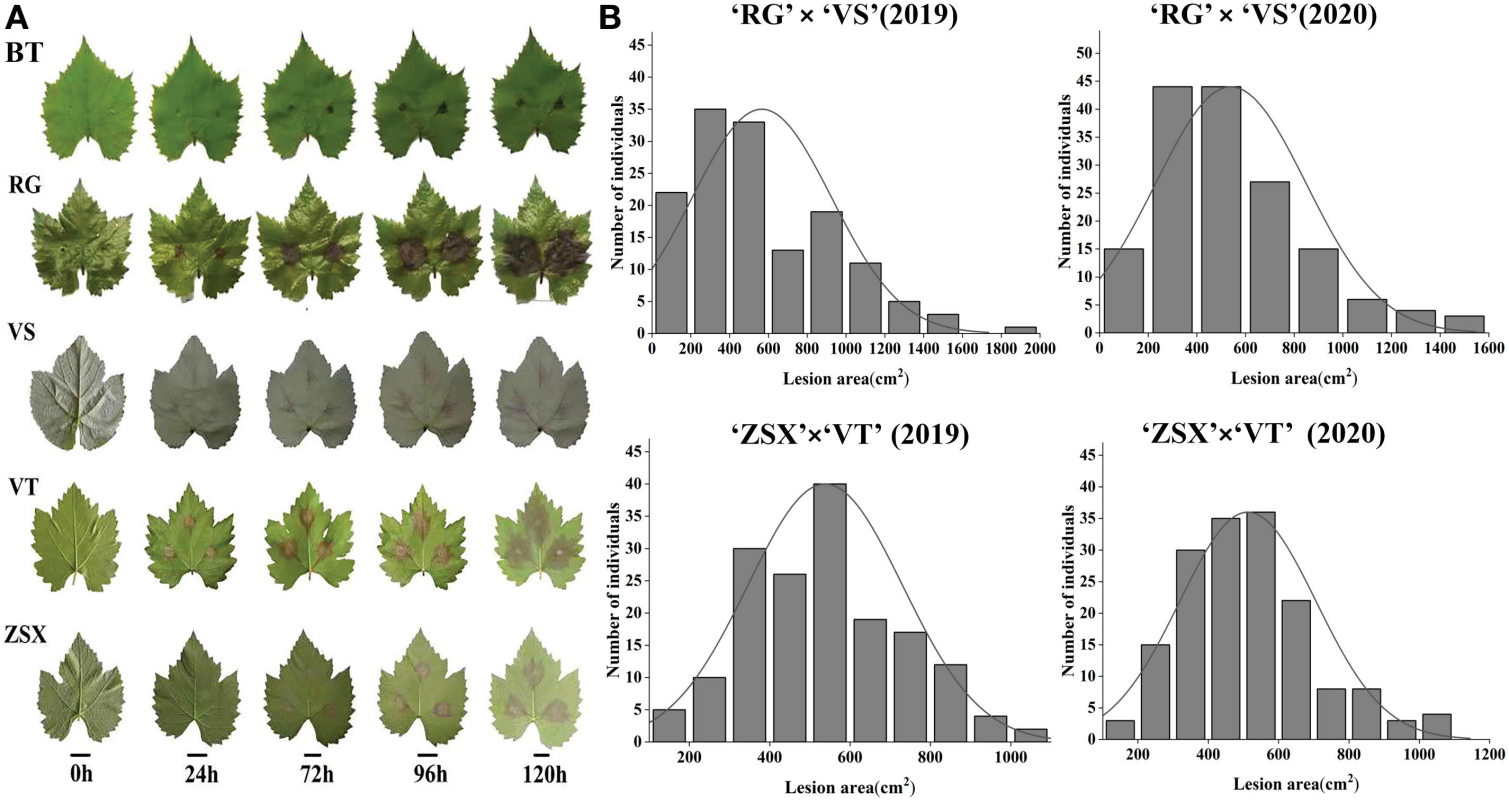
Grapevine gray mold resistance identification of five grape cultivars and two hybrid populations. **(A)** Gray mold lesion area identification of five grape cultivars. ‘BT,’ ‘RG,’ ‘VS,’ ‘ZSX,’ and ‘VT’ represent grape cultivars ‘Beta,’ ‘Red Globe,’ ‘Venus seedless,’ ‘Zhuosexiang,’ and’Victoria,’ respectively. **(B)** Gray mold lesion area distribution of two hybrid populations in 2019 and 2020.

Gray mold resistance identification of five grape cultivars, ‘RG,’ ‘VT,’ ‘ZSX,’ ‘VS,’ and ‘BT,’ at different infection stages was evaluated based on the lesion area (**Figure 1A**). Among these five cultivars, ‘BT’ showed the highest resistance to gray mold infection, and ‘ZSX’ also showed higher resistance compared with the other three cultivars. Furthermore, 176 hybrid progenies of ‘RG’ × ‘VS’ and 177 hybrid progenies of ‘ZSX’ × ‘VT’ were identified for gray mold resistance in 2019 and 2020; the results of these two hybrid progenies showed continuous variation (**Figure 1B**; **Table S2**). These results indicated that gray mold resistance in grapevine was a typical quantitative trait controlled by multiple genes.

### 3.2 Gene function annotation and differential expression analysis

**Figure 2:**
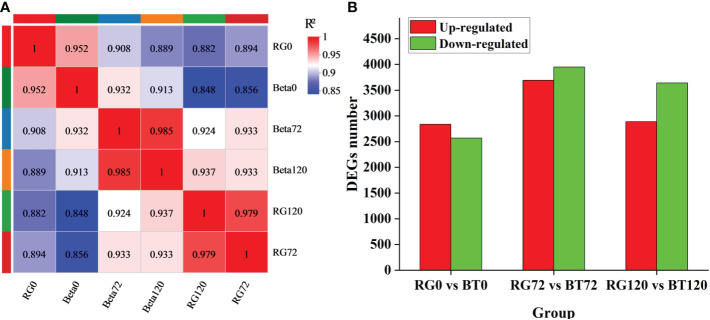
Transcriptome and differentially expressed gene analysis. **(A)** Pearson’s correlation coefficient analysis for gene expression levels between each sample. **(B)** Differentially expressed gene statistics in RG0 vs. BT0d, RG72 vs. BT72, and RG120 vs. BT120.

To identify candidate genes involved in grape gray mold resistance, we conducted RNA-seq for grapevine cultivars ‘RG’ and ‘BT’ at 0, 72, and 120 h after infection. After removing low-quality reads and adapters, a total of 124.20 Gb Clean Data were harvested and retained for further analysis. The average clean data of each sample were 6.17 Gb and have been uploaded to NCBI Sequence Read Archive (SRA) with the Accession Number PRJNA788159. The clean data were assembled using StringTie software. In total, 50,817 annotated transcripts from 42,416 gene loci were obtained through aligning with Swiss-Prot, GO, Kyoto Encyclopedia of Genes and Genomes (KEGG), and Pfam databases by using BLAST and HMMER software (**Tables S3, S4**). The FPKM value that was calculated by the comparison of sequenced reads with obtained RNA-seq database represents the expression of each transcript (**Table S4**). To confirm the reliability and rationality of the experiment, we calculated the Pearson’s correlation coefficients for all gene expression levels between each sample and reflected these coefficients in the form of a correlation matrix map (**Figure 2A**). A total of 5,407 genes were differentially expressed in RG0 vs. BT0 {|[log2 (fold change)]| >1 and adjusted P < 0.05} after differential expression analysis, among which 2,838 were upregulated and 2,569 were downregulated; 7,642 genes were differentially expressed in RG72 vs. BT72, among which 3,693 were upregulated and 3,949 were downregulated; 6,529 genes were differentially expressed in RG120 vs. BT120, among which 2,887 were upregulated and 3,642 were downregulated (**Figure 2B**).

### 3.3 Gray mold resistance gene discovery based on QTL mapping

**Figure 3:**
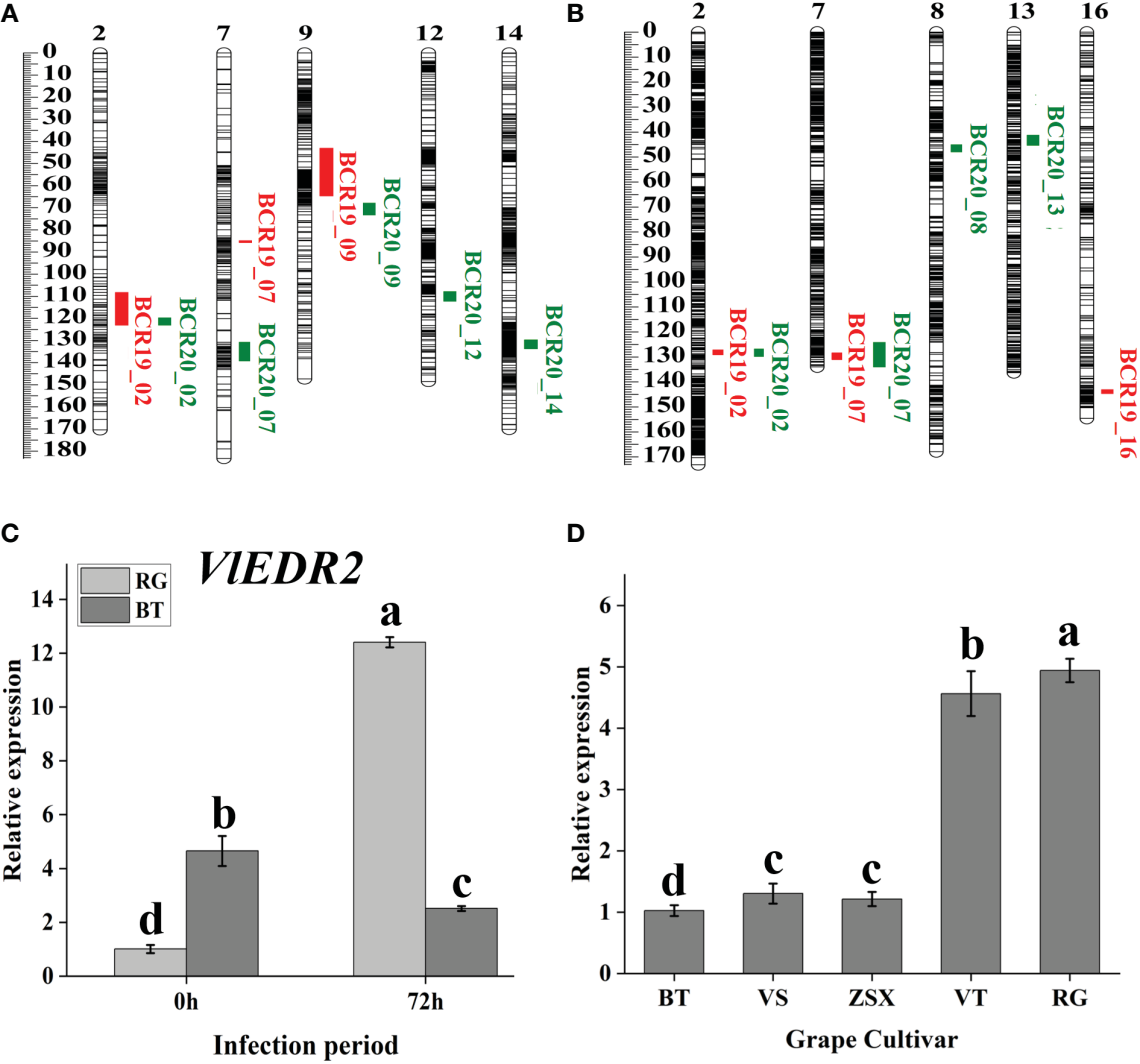
Candidate gray mold resistance gene discovery based on QTL mapping. **(A, B)** Gray mold resistance QTL mapping based on the hybrid population ‘ZSX’ × ‘VT’ and ‘RG’ × ‘VS’. **(C)** Cluster heat map of gene expression involved in the common interval of stable QTL. **(D)** qRT-PCR analysis of candidate gray mold resistance gene *VlEDR2* at different infection periods. Light-gray bars represent cultivar ‘RG,’ and dark-gray bars represent cultivar ‘BT.’ Error bars represent the standard deviation of three biological replicates. Lowercase letters on the bar chart represent significant differences between the two cultivars and different developmental stages according to Duncan’s multiple range test at P < 0.05.

Based on the gray mold identification of hybrid offspring in 2019 and 2020 and our constructed genetic linkage maps, we conducted QTL mapping to further discover the candidate genes related to grape gray mold resistance (**Figure 3**; **Table 1**). Eight potential QTL related to grape gray mold resistance were identified on LG2, LG7, LG9, LG12, and LG14 in the integrated map of ‘ZSX’ × ‘VT’ (**Figure 3A**), and the phenotypic variation they explained ranged from 6.70% to 16.50%. Seven potential QTL were identified on LG2, LG7, LG8, LG13, and LG16 in the integrated map of ‘RG’ × ‘VS’ (**Figure 3B**), and the phenotypic variation they explained ranged from 4.40% to 15.10%. Interestingly, four potential QTL on LG2 were detected stable in the two integrated maps in 2019 and 2020, and these stable QTL accounted for 6.86%–13.70% of the phenotypic variation in the gray mold resistance. Two potential QTL on LG7 were detected stable in the integrated map of ‘RG’ × ‘VS’ in 2019 and 2020. These stable QTL accounted for 4.40%–11.40% of the phenotypic variation in the gray mold resistance.

**Table 1:** Gray mold resistance QTL mapping based on the hybrid offspring of ‘ZSX’ × ‘VT’ and ‘RG’ × ‘VS’.

Population	Year	LG	LOD threshold	Peak LOD	Peak location	PEV (%)	Confidence Interval (CI)
‘ZSX’ × ‘VT’	2019	2	3	3.95	123	6.86	9733811-14339399
		7	3	3.65	85.6	16.50	11039523-11495736
		9	3	4.93	64.6	12.80	9405408-14429438
	2020	2	3	3.29	121	7.00	12217468-14339399
		7	3	4	134	6.70	16739137-18634132
		9	3	4.13	68.7	12.84	15860950-17484187
		12	3	3.04	109.1	9.70	20219297-21021838
		14	3	3.14	131.6	7.30	23813814-26365608
‘RG’ × ‘VS’	2019	2	3	4.01	128.67	12.9	13598944-13740048
		7	3	3.17	130	11.40	20767619-20873218
		16	3	3.01	146	15.10	19131784-19424306
	2020	2	3	3.3	127.9	13.70	13516138-13852989
		7	3	3.74	125.3	4.40	19689906-21022368
		8	3	3.57	47.3	7.00	8143755-8403936
		13	3	4.92	46.3	6.33	6414748-7273900

**Figure 4:**
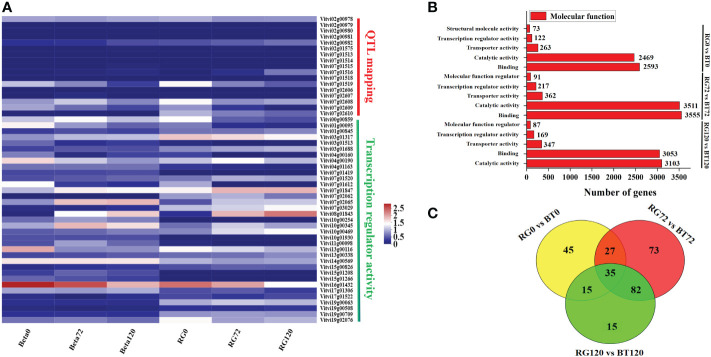
Differentially expressed structural gene and transcription factor analyses based on QTL mapping and GO enrichment. Analysis for grape cultivars ‘RG’ and ‘BT’ at different infection periods. **(A)** Cluster heat map of structural gene and transcription factor expression. **(B)** Number of differentially expressed genes in “Molecular function” catalog at different infection periods. **(C)** Common differentially expressed transcription factor identification in RG0 vs. BT0d, RG72 vs. BT72, and RG120 vs. BT120.

**Figure 5:**
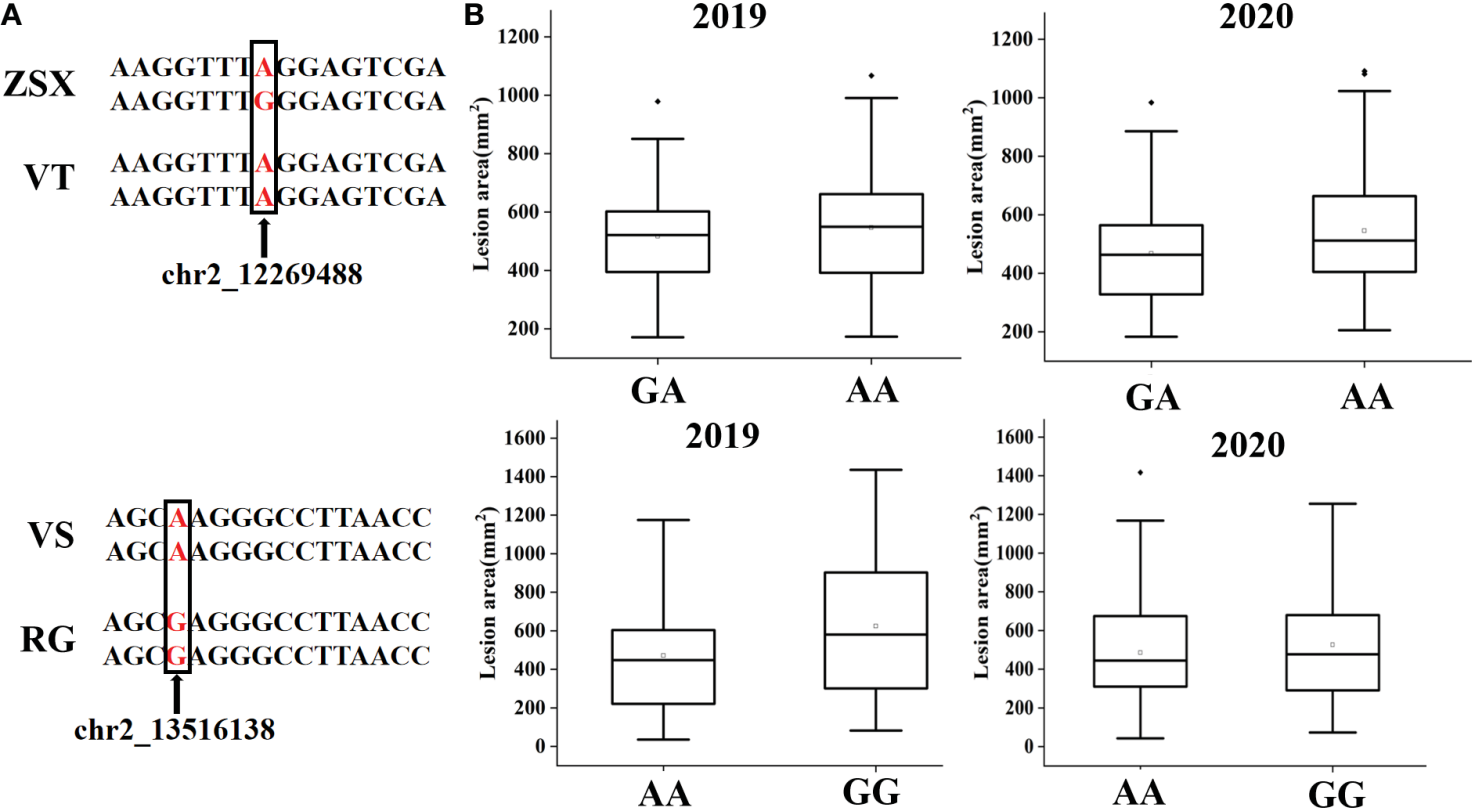
Distributions of grape hybrid gray mold lesion area according to the markers chr2_12269488 and chr2_13516138 in the population of ‘ZSX’ × ‘VT’ and ‘RG’ × ‘VS.’ **(A)** Base information of markers chr2_12269488 and chr2_13516138 in different cultivars and the flanking sequence. **(B)** Gray mold lesion area distribution of F1 progeny from the population of ‘ZSX’ × ‘VT’ and ‘RG’ × ‘VS’ in different years.

According to the QTL mapping, the common physical intervals of stable QTL were 13598944-13740048 in chromosome 2 and 20767619-20873218 in chromosome 7. In this study, we majorly focused on the candidate genes that were involved in the common intervals, and finally, 17 genes were discovered (**Table S5**). After analyzing the differential expression of the selected genes in different comparison groups (RG0 vs. BT0, RG120 vs. BT120, RG0 vs. RG120, and BT0 vs. BT120) with |[log_2_FC]| >1 and adjusted P < 0.05 (**Figure 4A**; **Table S3**), we finally screened the candidate gene *Vitvi02g00982* that annotated as enhanced disease resistance 2 (*VlEDR2*) for further analysis (**Figure 3C**). The results showed that the expression of *VlEDR2* in ‘RG’ was significantly upregulated after gray mold infection, and the expression level in ‘BT’ was significantly downregulated; the expression level of *VlEDR2* in ‘RG’ was significantly higher than that in ‘BT’ at 72 h (P < 0.05). After that, the expression level of *VlEDR2* in grapevine cultivars ‘VT,’ ‘ZSX,’ and ‘VS’ was also identified (**Figure 3D**). The result showed that the expression of *VlEDR2* in sensitive cultivars was significantly higher than that in resistant cultivars (**Figure 3D**). The Kruskal–Wallis test was employed to analyze the relationships between the phenotypic values and genotypes of the markers on LG2 and LG7, which showed a significant correlation at P < 0.05. Markers chr2_12269488 and chr2_13516138 were most significantly linked to gray mold resistance in the population of ‘ZSX’ × ‘VT’ and ‘RG’ × ‘VS’ according to the Kruskal–Wallis test (**Figure 5**). These two markers were located at 12,269,488 bp and 13,516,138 bp on chromosome 2. Raw sequencing data related to these markers were analyzed, and the nucleotides were A/A in ‘VT,’ G/A in ‘ZSX,’ A/A in ‘VS,’ and G/A in ‘RG.’ Progeny carrying A/A in the population of ‘ZSX’ × ‘VT’ generally showed susceptible phenotypes, and the average lesion area of A/A individuals in 2019 and 2020 was 554.7 mm^2^ and 530.2 mm^2^, respectively; whereas G/A individuals generally showed resistance, and the average lesion area of G/A individuals in 2019 and 2020 was 517.6 mm^2^ and 467.7 mm^2^, respectively. Progeny carrying G/G in the population of ‘RG’ × ‘VS’ generally showed susceptible phenotypes, and the average lesion area of G/G individuals in 2019 and 2020 was 624.1 mm^2^ and 525.3 mm^2^, respectively; whereas A/A individuals generally showed resistance, and the average lesion area of A/A individuals in 2019 and 2020 was 470.9 mm^2^ and 484.9 mm^2^, respectively.

### 3.4 Transcription factor discovery related to *VlEDR2* regulation

In our study, we selected a candidate grape gray mold-sensitive gene *VlEDR2* based on QTL mapping (**Figure 3**). To further identify transcription factors involved in *VlEDR2* regulation, we conducted GO enrichment analysis for these DEGs in the group of RG0 vs. BT0, RG72 vs. BT72, and RG120 vs. BT120. A total of 122, 217, and 169 genes in “Transcription regulator activity” cataloged under “Molecular function” were discovered, respectively (**Figure 4B**). Finally, 35 DEGs were selected for their significantly different expression in RG0 vs. BT0, RG72 vs. BT72, and RG120 vs. BT120 (**Figures 4A–C**; **Table S6**), among which 21 annotated genes were from ERF, MYB, MAD-box, NAC, and WRKY families, and we majorly focused on these 21 transcription factors.

**Figure 6:**
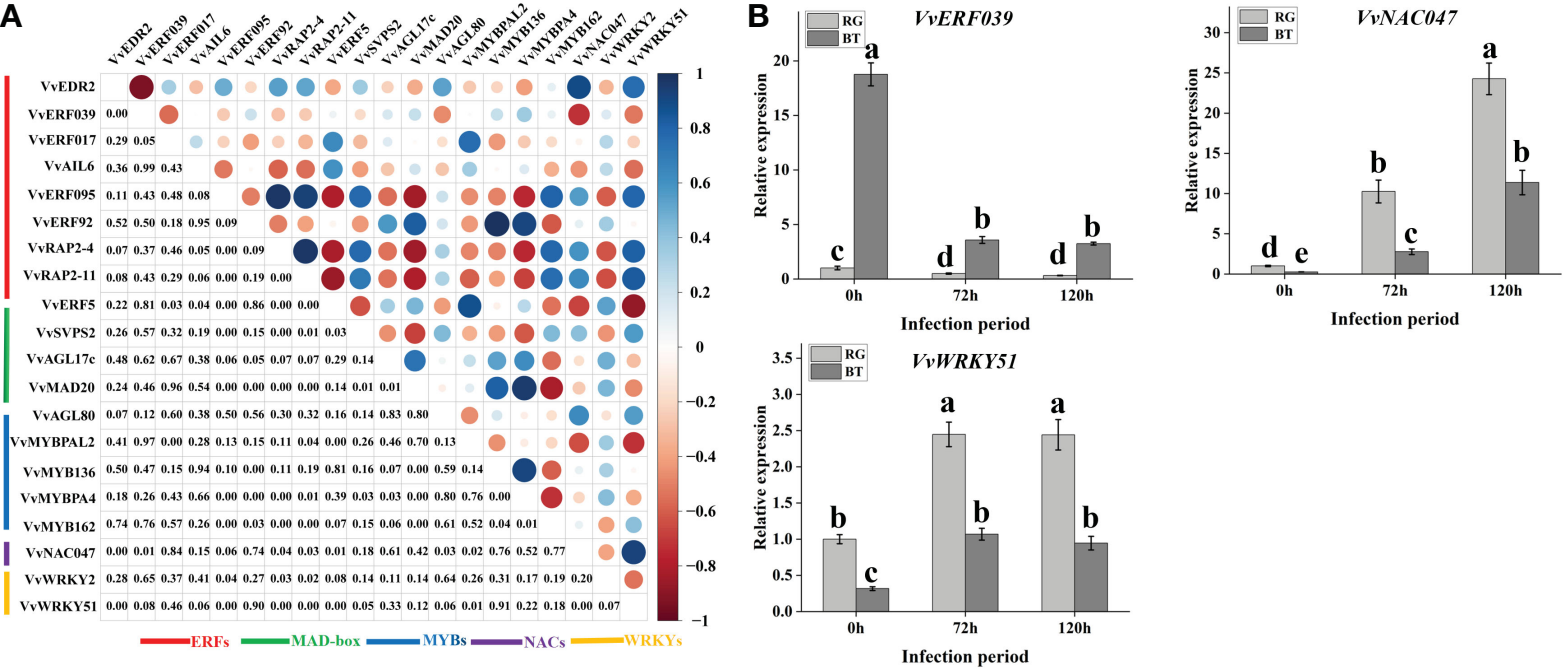
Candidate transcription factor filter related to *VlEDR2* expression and grapevine gray mold resistance. **(A)** Correlation analysis of transcription factors from different families with candidate gray mold resistance gene *VlEDR2* at different infection periods. **(B)** qRT-PCR analysis of candidate gray mold resistance transcription factors at different infection periods. Light-gray bars represent cultivar ‘RG,’ and dark-gray bars represent cultivar ‘BT.’ Error bars represent the standard deviation of three biological replicates. Lowercase letters on the bar chart represent significant differences between the two cultivars and different developmental stages according to Duncan’s multiple range test at P < 0.05.

To further select relevant transcription factors related to *VlEDR2* expression, the FPKM values of these 21 transcription factors and *VlEDR2* at 0 and 72 h were used to conduct the correlation analysis (**Figure 6A**). Finally, three candidate transcription factors, *VlERF039* (Vitvi00g00859), *VlNAC047* (Vitvi08g01843), and *VlWRKY51* (Vitvi07g01847), from ERF, NAC, and WRKY families that showed a significant correlation (P < 0.05) with the expression of *VvEDR2* were selected. The qRT-PCR verification showed that *VlERF039* was repressed in ‘RG’ and ‘BT’ after gray mold infection, and the expression level in ‘BT’ was significantly higher than that in ‘RG.’ *VlNAC047* and *VlWRKY51* that showed a positive correlation with *VlEDR2* were also identified. The expression of *VlNAC047* and *VlWRKY51* was induced in ‘BT’ and ‘RG,’ and the expression level of these two candidate genes in ‘RG’ was significantly higher than that in ‘BT’ (**Figure 6B**).

## 4 Discussion

### 4.1 The formation of heterobeltiosis and lower QTL effect

In this study, some individuals from our two constructed hybrid offspring showed higher gray mold resistance than their parent cultivar ‘ZSX’ and ‘VS.’ The additive effects of several desired dominant alleles or the combined effect of different alleles at the same gene locus, or a combination of both, may have formed heterobeltiosis, and the genetic differences between parents are the primary cause of it. According to heterobeltiosis, we can screen for superior parents and predict the heterosis of parental combinations. In our study, a total of 12 individuals that showed higher gray mold resistance than their resistant parents from the hybrid progenies of ‘RG’ × ‘VS’ and ‘ZSX’ × ‘VT’ were identified, and transgressive offspring in our study provided important grape gray mold resistance resources, and they can also be used as material for underlying genetic and molecular mechanisms of grape gray mold resistance.

QTL mapping and candidate gene discovery of grapevine gray mold resistance are important for grape breeding. In our study, we discovered two stable QTL related to gray mold resistance that were located on linkage groups LG2 and LG7. While the phenotypic variance of these QTL ranged from 6.86% to 13.70% on LG2 and 4.40% to 11.40% on LG7, the smaller QTL effect may be due to the quantitative nature of the host resistance, and according to the Beavis effect, when the sample size was small, the QTL effect would be greatly inflated, and the larger the sample size, the smaller the QTL effect and the closer to the true value (Beavis, 1994; Göring et al., 2001; Slate, 2013).

### 4.2 Discovery of structural genes related to gray mold resistance

Structural genes related to gray mold resistance were majorly involved in the pattern recognition receptor (PRR)-triggered immunity (PTI) that could mediate gray mold resistance through recognizing pathogen-associated molecular patterns (PAMPs) and host damage-associated molecular patterns (DAMPs), such as chitin elicitor receptor kinase 1 (CERK1), LysMdomain-containing glycosylphosphate ethylinositol-anchored protein 2 (LYM2), and wall-associated kinase 1 (WAK1), and polygalacturonidase-inhibiting proteins (PGIPs) *Botrytis*-induced kinase 1 (BIK1), MPK2/3/6, PAD3, and *Arabidopsis* histidine kinase 5 (AHK5) (Miya et al., 2007; Qiu et al., 2008; Ren et al., 2008; De Lorenzo et al., 2011; Eckardt, 2011; Galletti et al., 2011; Birkenbihl et al., 2012; Pham et al., 2012; Faulkner et al., 2013; Zhang et al., 2014; Guan et al., 2015; Liu et al., 2015). In grapevine, some structural genes related to gray mold resistance have also been reported (Agüero et al., 2005; Agudelo-Romero et al., 2015; Jiao et al., 2015; Rubio et al., 2015; Wang Y. et al., 2017; Wan et al., 2021), but most of these genes were selected through either previous research or transcriptome analysis based on two different gray mold resistance cultivars. In our study, we firstly conducted grape gray mold resistance QTL mapping supplemented by transcriptomic analysis, and finally, a new candidate resistance gene *VvEDR2* was selected. Based on previous research, *EDR* played a negative role and the *edr* mutants display high resistance (HR)-like lesions in response to a pathogen attack stimulus such as powdery mildew in plant that is involved in the salicylic acid (SA) defense pathway (Frye and Innes, 1998; Tang et al., 2005a; Tang et al., 2005b; Tang et al., 2006). Moreover, some studies have also shown the SA-independent phenotype of *EDR2* that is involved in hypersensitivity to ethylene-induced senescence, implicating *EDR2* in the regulation of senescence and defense signaling (Frye et al., 2001; Tang et al., 2005b). In our study, we preliminarily identified the potential role of *VlEDR2* in negatively regulated grapevine gray mold resistance, and this discovered resistance gene will provide new reference for the research on grapevine gray mold resistance.

### 4.3 Candidate transcription factors involved in the regulation mechanism of gray mold resistance

Many reports have shown the role of ERFs in plant gray mold resistance, such as *RAP2.2*, *ORA59*, *ERF1*, *ERF5*, and *ERF6* in *Arabidopsis thaliana*; overexpression of these genes could enhance the resistance to gray mold through binding to GCC-box elements of defense marker gene *PDF1.2* and promoting its expression in jasmonic acid (JA) and ethylene (ET) signaling pathways (Berrocal-Lobo et al., 2002; Pre et al., 2008; Zarei et al., 2011; Moffat et al., 2012; Zhao et al., 2012). In tomato, silencing of *SlERF.A1*, *SlERF.A3*, *SlERF.B4*, or *SlERF.C3* resulted in increased susceptibility to *B. cinerea* (Ouyang et al., 2016). In grapevine, overexpression of *VqERF072*, *VqERF112*, *VqERF114*, and *VaERF20* in *A. thaliana* could also enhance the resistance to *B. cinerea* in JA and ET signaling pathways (Wang et al., 2018a; Wang et al., 2020). WRKY TFs could also regulate gray mold resistance through activating the expression of structural genes involved in SA and JA signaling, such as *LrWRKY4*, *LrWRKY12*, and *LrWRKY39* in *Lilium* (Cui et al., 2018; Fu et al., 2022), *SlDRW1* and *SlWRKY46* in tomato (Liu et al., 2014; Shu et al., 2021), *RcWRKY41* in rose (Liu et al., 2019), and *VqWRKY52* in grapevine (Wang X. et al., 2017). Moreover, TFs from the MYB family could also play positive and negative regulatory roles in gray mold resistance, such as *RcMYB84*, *RcMYB123*, and *MYB108* in JA signaling pathway (Mengiste et al., 2003; Ren et al., 2020; Cui et al., 2022) and *MYB72* in induced systemic resistance signaling pathway (Van der Ent et al., 2008). *MYB46* negatively mediated gray mold resistance through repressing the synthesis of cellulose synthases (Ramirez et al., 2011), and *BjMYB1* positively regulated gray mold resistance through activating the expression of *BjCHI1* (Gao and Zhao, 2017). In grapevine, the interaction of *VaERF16* and *VaMYB306* could increase the expression level of *VaPDF1.2* and then enhance gray mold resistance (Zhu et al., 2022). In our study, based on the expression pattern of *VlEDR2*, we screened out a new candidate ERF gene *VlERF039* and WRKY gene *VlWRKY51*, while their potential possibility in regulating the expression of *VlEDR2* and grape gray mold resistance still needs a deep exploration. Moreover, NAC gene *VlNAC047* was also discovered, and until now, there was no report focused on its function in gray mold resistance; this discovery can provide a new insight on transcriptional regulation mechanisms of grape gray mold resistance.

## 5 Conclusion

Based on QTL mapping and transcriptome analysis, we discovered one structural gene, *VlEDR2* (Vitvi02g00982), which may play a negative role in grapevine resistance to gray mold. Moreover, three potential transcription factors including *VlERF039* (Vitvi00g00859), *VlNAC047* (Vitvi08g01843), and *VlWRKY51* (Vitvi07g01847) that may influence the expression of *VlEDR2* and grapevine gray mold resistance in positive and negative ways were also discovered. The candidate genes identified in our study will provide an important reference for research into grapevine gray mold resistance mechanisms and breeding in grape species.

## Data availability statement

The original contributions presented in the study are publicly available. This data can be found here: NCBI, PRJNA788159.

## Author contributions

YG, KS, and YZ contributed to experimental design, KS and WZ performed the experiments and KS wrote the article. KS and WZ performed grapevine gray mold identification and KS, HL, and CJ contributed to data analysis. All authors contributed to the article and approved the submitted version.
